# Causes or Cures: What makes us think of attention issues as disorders?

**DOI:** 10.1016/j.newideapsych.2023.101008

**Published:** 2023-04

**Authors:** Andreas De Block, Siegfried Dewitte, Kristien Hens

**Affiliations:** a Centre for Logic and Philosophy of Science, Kardinaal Mercierplein 2 - box 3200, 3000, Leuven, Belgium; b Department of Marketing, Leuven, Naamsestraat 69 - box 3545, 3000, Leuven, Belgium; c Department of Philosophy, Rodestraat 14, 2000, Antwerpen, Belgium

**Keywords:** Concepts of disease, Experimental philosophy, Philosophy of medicine, Vignettes

## Abstract

Are attention issues disorders or not? Philosophers of medicine have tried to address this question by looking for properties that distinguish disorders from non-disorders. Such properties include deviation of a statistical norm, a loss of function or experienced suffering. However, attempts at such conceptual analysis have not led to a consensus on the necessary and sufficient conditions for the application of the concept of disorder. Recently, philosophers have proposed an experimental approach to investigate in which circumstances people think a specific concept is applicable. Here we present a quantitative vignette study investigating whether disorder attribution depends on the perceived cause and the perceived type of treatment for an attention problem. The results of our study indicate that the attribution of a disorder decreased when the attention problem was understood as caused by bullying (social environmental cause) or by an accident (non-social environmental cause) rather than a genetic cause. When prescribed a pill, attention problems were considered a disorder to a larger extent than when the child was prescribed an environmental treatment. Our study also suggests that whereas successful environmental treatments will not necessarily decrease the disorder attribution, successful pharmacological treatments will decrease the likelihood that a person is thought to still suffer from a disorder after receiving the treatment.

## 1 Introduction

Both scientists and laypeople are deeply divided over whether particular attention-related challenges are genuine disorders (Conrad & Bergey, 2014; Harwood et al., 2017; Malacrida, 2004; Whitley, 2021). The proponents of considering attention challenges as disorders emphasize the benefits of such medicalization. In contrast, the opponents stress how such medicalization negatively affects the well-being of those with problems concentrating. However, the debate is not just about a cost-benefit analysis of medicalization. There also seems to be an issue that centres around the (in)correct application of the disorder concept to particular attention and concentration problems. Are there attention states or mechanisms that are genuinely disordered, and if so, in what sense do they differ from healthy or normal states or mechanisms? A philosophical analysis of the disorder concept may seem helpful in addressing these questions. However, philosophers propose and defend widely divergent accounts of disorder (Faucher & Forest, 2021; Kingma, 2010). Some think the concept cannot be successfully analyzed with the help of traditional philosophical tools (Ereshefsky, 2009). Moreover, these analyses rely heavily on what philosophers think are the intuitions of competent speakers of their linguistic community but do not build on a systematic study of these intuitions (De Block & Hens, 2021; Machery, 2017; Veit, 2021b).

In this paper, we propose a systematic study of people’s medical intuitions. This study aims at improving our understanding of the factors that undergird the medicalization or demedicalization of attention problems. Using vignettes, we explored how perceived causes and types of treatment of attention problems influence the application of the disorder concept. In section 2, we will present the theoretical background for our study, sketching both the stakes in the medicalization debate, and the reasons why traditional conceptual analysis is unlikely to resolve this debate. In section 3, we describe the method used in this study. In section 4, we report the results of our study. In section 5, we interpret the findings and discuss their implications for the philosophical and scientific study of medicalization.^1^

## 2 Theoretical background

Early research on medicalization tended to be qualitative and based on case studies (Conrad, 1992). However, over the last two decades, cognitive science has offered a series of quantitative and systematic studies on how disease or disorder labels affect individuals. Overall, these studies give a nuanced picture, indicating that such labels are a mixed blessing (Haslam & Kvaale, 2015; Kvaale, Gottdiener, & Haslam, 2013; Lebowitz & Appelbaum, 2019): on the one hand, labelling a bodily condition, a mental state or behaviour as a disorder can dehumanize and stigmatize; on the other hand, these labels also tend to have exculpatory effects (Kvaale, Haslam, & Gottdiener, 2013; Lin, 2017).

There are other ambivalent effects as well, as is illustrated by the debate on the inclusion of complex bereavement disorder, or complex grief, in diagnostic manuals (American Psychiatric Association, 2013; Parkes, 2020). Scholars favouring its inclusion have argued that this would give sufferers (more accessible) access to treatment specific for grief, if they need it, rather than being prescribed antidepressant medication (Prigerson et al., 2022). Although this may stimulate research into treatment for complex grief, pathologizing normal grief might lead to suboptimal research and clinical resource allocation (Horwitz & Wakefield, 2007). The literature on the medicalization of concentration problems highlights similar issues (Kristjánsson, 2009; Lusardi, 2019) and suggests that it matters significantly whether these problems are labelled disorders.

Philosophers of medicine have long tried to come to the rescue and lay down criteria to delineate disorders from non-disorders (Hofstad et al., 2020). Traditionally, the focus has been on delineating disorder from non-disorder (or disease from non-disease). These attempts have resulted in naturalist, normativist and hybrid definitions. Naturalists, such as Christopher Boorse, state that what is a disorder can be grounded in (biological) facts, although he does not discuss much actual biology. According to Boorse's biostatistical theory (BST), a disorder is defined by the abnormal functioning of an organism or part of an organism (Boorse, 1977). In his view, the ‘abnormal’ refers to the fact that the part's contribution to the organism's biological goals of survival and reproduction is statistically atypical. Normativists, in contrast, think that disorder judgments are purely value judgements (Cooper, 2002). Hybrid accounts combine elements of naturalism and normativism and argue that value judgments must accompany scientific judgments to draw the line between health and disorder. Jerome Wakefield has advocated such a hybrid analysis. According to him, a condition is a disorder if and only if the condition is both dysfunctional and harmful (Wakefield, 1992).

Sophisticated philosophical analyses of the disorder concept are probably somewhat relevant for medicalization debates. Nevertheless, there are also reasons as to why the conceptual analysis of disorder will not have the final word. First, philosophers of medicine have had little influence on medical classifications or definitions of disorder in widely used medical handbooks (Sholl & Okholm, 2021). Second, there is widespread dissensus on the correct account of disorder, and different philosophical accounts of disorder lead to different assessments of (de)medicalization attempts (De Block, 2020). For instance, according to Boorse's definition, homosexuality satisfies the conditions of being a disease, whereas Wakefield holds that it is not a disorder. Thirdly, there is much disagreement within the different approaches. For instance, naturalists disagree on which biological facts matter for our disease judgements (Veit, 2021a). Fourth, whereas traditional conceptual analysis relies on what philosophers think are the intuitions of competent speakers of their linguistic community about what counts as a disorder and what counts as a healthy condition, these analyses tend to neglect observed variations in such intuitions and the causes of these variations. In other philosophical subdisciplines, this neglect has been addressed by the rise of experimental philosophy. In philosophy of medicine, however, experimental work is relatively scarce (De Block & Hens, 2021; Veit, 2021b).

In our view, the main contribution of such experimental work is not to provide the necessary and sufficient conditions for the correct application of the disease or disorder concept (Machery, 2017). Also, experimental studies like ours should largely be irrelevant for nosological decisions. The relevant epistemic aims (such as construct validity) and non-epistemic aims (such as inclusivity) of these decisions (Solomon, 2015; Tekin, 2019) are not served by including data on the views of an undifferentiated public on mental conditions. What experimental work does offer, though, is an investigation of how people conceptualize disorders(Aftab, 2021). This approach fits well within what Joshua Knobe has called the cognitive science program of experimental philosophy (Knobe, 2016).

While cognitive science has researched some of the *effects* of medicalization, it has so far paid little attention to factors that facilitate or hamper medicalization. This is a significant lacuna in the literature. Sociologists have researched how the lobbying by pharmaceutical companies, patient organizations and insurance companies has led to medicalizing specific behavioural variants and tendencies (Conrad & Leiter, 2004; Frances, 2014; Hartley, 2003). However, we still lack a good understanding of the psychological mechanisms that make attributing disease or disorder labels more likely. Such understanding is essential because it can explain, for instance, differences in the success of lobbying. More generally, the findings of experimental philosophy of medicine seem relevant for health communication and (other) educational purposes since the success of such initiatives depends on considering what people tend to think. For instance, health communication initiatives could profit from understanding which properties make it (more) likely that disorder labels will be applied and which properties diminish the likelihood of such application.

One candidate for such a property is ‘suffering’. Of course, that does not entail that all suffering is pathological (Wakefield, 2007). Nevertheless, it seems likely that emphasizing the mental or physical suffering that accompanies a particular condition increases the chances that it will be considered a disorder (Bergner & Bunford, 2017). Less trivial is the idea that the causes of a condition or behaviour may also impact our disorder intuitions. However, there is some evidence that points in that direction. For instance, in the 19th century, degeneration theories played an important role in pathologizing unusual sexual desires and behaviors (Rimke & Hunt, 2002). Likewise, in an interview on the results of a genome-wide association study of ADHD, one of the authors claimed that “especially for ADHD, which is by some still not believed to be a real disorder, identifying the underlying genes and biological mechanisms is of great importance.” (*Researchers have found the first risk genes for ADHD* *- New insight into the biology behind ADHD*, n.d.) Similarly, it has recently been argued that symptoms of functional neurological disorders, traditionally labelled as ‘hysteria’, are not ‘faked’ *because* they have a neurobiological basis (Madva et al., 2019, italics ours). This suggests that (perceived) causes impact people's attribution of disorder status.

Interestingly, Ahn and colleagues found a link between (causal) assumptions about mental disorders and specific forms of therapy. Clinicians tend to believe medication is more effective for (presumed) biologically-based mental disorders. In contrast, psychotherapy is seen as more effective for (presumed) psychosocially-based mental disorders (Ahn et al., 2009-3). However, the relation between perceived cause and beliefs in effective treatment may be more complicated. First, an effective pharmacological or surgical intervention may also suggest a particular cause, say a genetic cause. Secondly, the success of a particular treatment might contribute to the belief that the treated person indeed was suffering from a ‘real disease’. Critics of ‘Big Pharma’ and ‘their disease mongering’ have often argued that “before you sell a drug, you have to sell a disease” (Lane, 2007). It does not seem too farfetched to think that it can also go the other way and that people can be strengthened in their belief that a condition is a ‘real disorder’ if it responds well to a pill or a surgical intervention (Browne, 2015). Here, we want to set the first step in that direction by studying how perceived causes and different types of therapies steer people's judgment on whether a concentration issue is a disorder or not. We put forward three hypotheses. We hypothesized that the designated cause of a symptom influences the assignment of disorder status, with genetic causes increasing the likelihood that specific atypical behaviour is seen as a disorder compared to environmental (social or non-social) causes. We hypothesized that the nature of the proposed treatment of a symptom influences the assignment of disease status, with pharmacological treatments increasing the perception of an atypical behaviour as a disorder as compared to environmental treatments. Finally, we hypothesized that the effectiveness of the proposed treatment in reducing the suffering influences the assignment of disorder status^2^ in such a way that after successful treatment of any kind, the respondents will be less likely to attribute an attention disorder to the treated person.

## 3 Method

Our study is an experimental vignette study. Vignettes are often used in experimental philosophy (Machery, 2017). They are generally considered to elicit relevant judgements (or 'intuitions') about realistic scenarios and allow for the manipulation and control of independent variables (Aguinis & Bradley, 2014).

### 3.1 Design and material

**Fig. 1:**
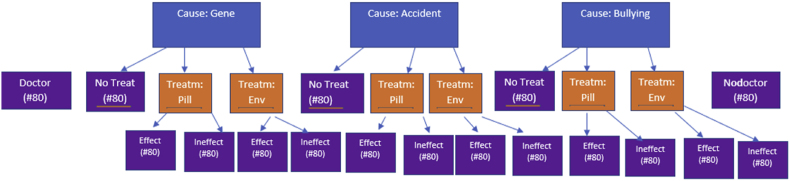
Design of the study.

*Phase 1*. We used a multilevel design for our vignettes (Fig. 1). Our general aim is providing insights in the (joint) effects of a stated cause and prescribed treatment on the perceived disorder status of an attentional problem. However, testing these effects with a two-factorial design cannot provide information about relative increases or decreases compared to a baseline without doctor visit. To address this problem, we added two baseline conditions, a baseline without doctor, and a baseline with doctor-condition, which solely served to tease apart the effect of the doctor visit per se. Text box 1 shows the vignette used. The ‘baseline without doctor’ condition (bold in Text box 1) introduced Jada as a six-year-old child with an attention problem. The ‘baseline with doctor’ repeated this information and added that Jada went to see a doctor for her problem (bold and italics in Text box 1). All other conditions also started with these two sentences but in the next sentences we added a full factorial design with the factors ‘cause’ and ‘treatment’. Specifically, participants in the experimental conditions were randomly assigned to one of three causes for the child's attention problems: the cause was either (1) a genetic variant, (2) an accident, or (3) bullying (see Text box 1, third sentence, normal font, one of three versions). Orthogonal to this factor stating the cause, we manipulated the treatment variable. The same participants were assigned to one of three treatment scenarios: one-third did not receive information about a treatment, but two-thirds (who got the underlined sentence in Text box 1) did. This underlined sentence only appeared in the two other thirds of the design. The second third learned that an environmental treatment had been prescribed. The final third learned that a pill had been prescribed (the two versions of the underlined sentence).

Text box 1 **the experimental vignette used.**

[phase 1] **Jada has had attention issues since she was very little. Moreover, she is easily scared by new situations. This makes her unhappy in the classroom.** *When she is six, her parents take her to the doctor.* The doctor tells them [Jada has a gene that/that the accident Jada had as a baby/her being bullied at school] is highly predictive for this kind of behavior. Jada is prescribed a [well-structured environment/pill]. From then on, she takes [this pill daily/is from then on educated in a well-structured environment].
[phase 2, only for those having received the underlined sentence] She is now nine years old and has [no/still] problems concentrating. She is [no longer/still] scared by new situations. She is [no longer/still] unhappy.

Alt-text: Text box 1

The dependent variables were the perceived disorder status, the plausibility of the cause and treatment, and the effectiveness of the intervention, all measured with 7-point Likert Scales. The plausibility analysis is beyond this paper's scope but can be acquired upon request. We then measured a series of control variables: gender of the participant, whether the participant was medically trained or not, whether the participant stated that they had been diagnosed with a developmental condition^3^ (Autism Spectrum Disorder/ADHD/Tourette/Dyspraxia/Dyslexia or other) and whether a medical consult was mentioned in the baseline. The exact wording of the questions and the vignettes can be found at the following URL: https://osf.io/4mzg7/files/osfstorage/607fdf0fc14695006c396c4e.

*Phase 2*. For all the participants who had learned about a treatment (6 conditions of the above design: the three causes times the two levels of treatment), we presented the same child three years later (at nine years old). Then we manipulated effectiveness. Half of those participants were randomly assigned, orthogonally to the two factors we manipulated in phase 1, to ‘effective treatment’ and a half to ‘non-effective treatment’. The same dependent variables were used for the second time.

So in sum, there were 2 (baselines) + 3 (no treatment, 3 causes) + 12 (treatment (2) x causes (3) x effectiveness (2) = 17 conditions, to which the 1408 participants were randomly assigned, leading to ca. 80 participants per condition (Fig. 1).

### 3.2 Sample

Participants were 1408 members of the Prolific platform, of which 31 did not give their informed consent (leaving us with 1377 participants). They all participated on 2021 April 25 or 26. There were 668 men, 699 women, four non-binary/third gender, and six who answered ‘preferred not to say’. 10% of the participants were between 18 and 24, 18% between 25 and 34, 18% between 36 and 44, 17% between 45 and 54, 22% between 55 and 64, 13% between 65 and 74, and 2% beyond 75 years old. Fifty-seven had a medical profession (4%). 92% of the participants had never been diagnosed with any neurodevelopmental condition. The most frequent diagnosis was Dyslexia (2%). The median duration of the study was 100 s, with a minimum value of 21 s. Six participants took longer than 15 min (900s) to complete the survey.

### 3.3 Analysis plan

The primary dependent variable is perceived disorder status (at age 6). Our general aim is to test the interplay between stated cause and the prescribed treatment on perceived disorder status. Before addressing this main question, we performed a preliminary analysis to evaluate to what extent the vignettes stating a cause lead to a different disorder status compared to the vignettes only stating the problem, or only the problem and the doctor visit (the two baseline conditions).

(1) Preparatory analysis. We perform an unifactorial GLM analysis with five levels: the baseline without seeing a doctor, the baseline with seeing a doctor, and the conditions with different causes (either genetic, an accident, or bullying) to test the cumulative effect of seeing a doctor and the specific cause of the problem on the perceived disorder status (phase 1 only). We corrected for multiple testing using the bonferroni rule. Specifically, we test seven contrasts: the pairwise differences between the two baseline conditions on the one hand and the three cause conditions on the other, plus the one contrast between the two baseline conditions, implying that we test at alpha = 0.05/7 = 0.007.
(2) Main analysis. We conduct a two-factorial GLM testing the effect of the cause (three levels, genes, environment, and bullying) and the proposed treatment (pills, environment, or no proposed treatment) to explore the cumulative effect of the treatment. In this analysis, we omit the baseline conditions (see preliminary analysis). We again conducted a bonferonni correction per factor (i.e. familywise). So for the cause, the treatment, and the interaction contrasts, we test at alpha 0.05/3 = 0.017. Note that this D.V. was measured before the factor effectiveness of phase 2 was applied.
(3) We then conducted an ANOVA with the same between-subject variables as in (2) and the factor 'effectiveness' added as an I.V., and with change in perceived disorder going from age six to age nine. This was only applied to the participants having received information about the treatment, and the effectiveness manipulation.
(4) We repeat all the analyses (1–3) inspired by a multiverse logic (Steegen et al., 2016), omitting the medical professionals, participants who ever received a neurodevelopmental diagnosis, and participants who took more than 15 min to answer the questions to assess the robustness of the findings to assess the robustness of the findings
(5) We repeat the analyses (1–3), controlling for gender and age to test the robustness of the findings and possible main effects of these control variables. Specifically, we added the categorical factors age and gender to our initial analysis and re-evaluated our findings from analysis (1), (2), and (3) and the possible effects of gender and age.

## 4 Results

(1) preliminary analysis

**Fig. 2:**
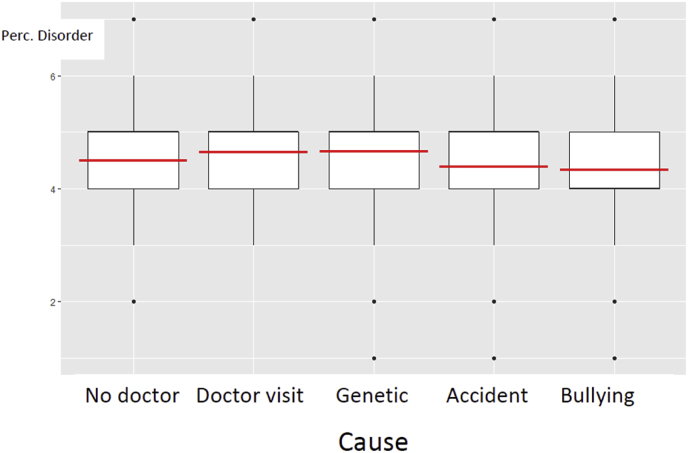
Disorder perception at age six as a function of whether or not they went to a doctor (no doctor, doctor) and which cause was identified (Genes, Accident, or Bullying).

**Fig. 3:**
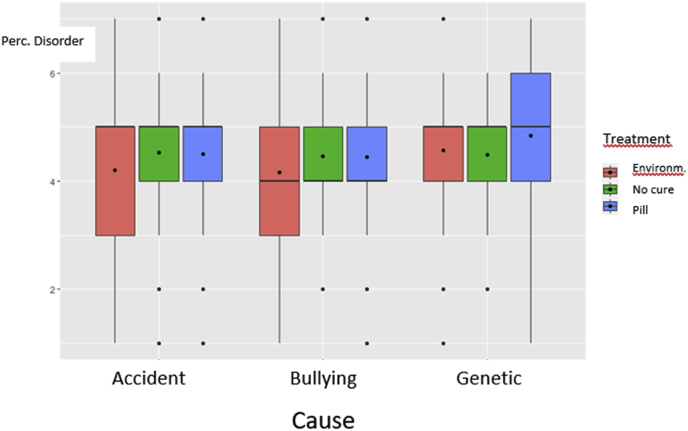
Perceived disorder at age six as a function of cause and proposed treatment.

Fig. 2 shows the differences in disorder perception between the ‘not seeing a doctor’-condition (“no doctor”), the ‘seeing a doctor’-condition (“doctor”), and three levels of diagnosed cause (“genetic”, “accident”, and “bullying”). An ANOVA showed that the differences were significant: F(4, 1372) = 4.40, p = .001. Note that the ‘no doctor’ and the ‘doctor’ baseline conditions have only 81 observations, so the contrasts with these conditions are statistically less powerful than the contrasts among the conditions with a cause in this analysis. Also note that in this analysis, we collapse over the type of treatment (see Fig. 3).

The no-doctor condition did not differ significantly from any other condition. The doctor-condition seemed to induce a stronger disorder status perception than when the cause was identified as an accident (t = −1.76, p = 0.08) or as bullying (t = −2.11, p = 0.04) but the differences didn't reach our alpha level of 0.007. Note that the omnibus test was significant due to the other contrasts, which we test in the next analysis. (2).

*Robustness checks.* Removing the excessively slow (longer than 15 min), the ones previously diagnosed with a mental disorder, and the medical professionals did not affect the conclusions (the general ANOVA: F(4,1211) = 4.28, p = 0.002). Gender and age did not affect the conclusions, but the perception of disorder increased with age (categorically coded): t = 2.82, p = 0.005.

(2) Two-factorial GLM on perceived disorder with cause and treatment as factors

The full factorial GLM analysis performed on the data of all participants except those in the two baseline conditions (n = 1215) revealed that mentioning the cause had an effect (F(2,1206) = 7.60, p < 0.001. When the cause was identified as an accident (M = 4.36, SD = 1.32) or as bullying (M = 4.31, SD = 1.32), it led to lower disorder status perceptions than when the cause was identified as a genetic cause (M = 4.70, SD = 1.33). Post-hoc contrast tests showed that both contrasts were significant (both contrasts p < 0.007, i.e. below the adapted alpha level). The proposed treatment also affected the perceived disorder status: F(2,1206) = 6.32, p = .002. When the child was prescribed an environmental treatment (M = 4.31, SD = 1.36), the attention problems were considered a disorder to a lesser extent than when prescribed a pill (M = 4.60, SD = 1.29). Post hoc contrast tests confirmed that this contrast was significant (p = .001, i.e. below the adapted alpha level). The control group (M = 4.47, SD = 0.93) did not significantly differ from either treatment condition, with the largest difference that being between environmental treatment and no treatment (diff: 0.18, p = .18). The interaction between the factors cause and treatment was not significant (F(4,1206) = 1.02, p = 0.40. Fig. 3 shows the boxplots of the six conditions.

The analysis of the trimmed dataset yielded virtually the same results. Adding gender and age as control variables did not affect the conclusions, but again, perceived disorder increased with age (b = 0.06, SE: 0.03, t(964) = 2.22, p = 0.03.

(3) Change in disorder perception as a function of cause, cure, and effectiveness

A full factorial ANOVA with the change^4^ in perceived disorder from age 6 to 9 as a dependent variable and cause, the treatment, and the effectiveness as independent variables led to significant results and revealed an (obvious) main effect of treatment effectiveness: b = −0.32, F(1, 960) = 62.27, p < 0.001. Except for the interaction between the treatment and its effectiveness (F(1, 960) = 2.57, p = 0.10) and the three-way interaction (F(1,960) = 1.03, p = 0.36) all other Fs < 1. Correcting for initial disorder perception had negligible effects. Removing those with a medical profession, prior diagnosis of mental disease, or spending more than 15 min (n = 848) made the interaction between treatment and its effectiveness marginally significant: F(1, 838) = 3.86, p = .05. Specifically, when the treatment was not effective, the treatment did not make much of a difference in the change from time 1 to time 2 regarding the perceived disorder status (Mpill = 0.05, SD = 0.69 vs Menvironment = −0.02, SD = 0.59). When the treatment was effective, however, the drop in disorder attribution from age six to nine was significant (see main effect), but it also depended on the treatment. The drop in disorder attribution was larger when the treatment was a pill than when it was environmental (Mpill = −0.61, SD = 1.29 vs Menvironment = −0.41, SD = 1.12).

## 5 Discussion

Disease and disorder are normatively important categories. We think disorders are bad things to have (Cooper, 2002), and generally, people prefer being healthy rather than ill. Furthermore, those who have a disease or a disorder are thought to deserve help and treatment (Biddle, 2016).

Our experiments indicate that whether respondents judge an attentional problem as a disorder is influenced by the perceived cause and the intervention proposed to resolve the problem. Moreover, the effect of perceived cause on disease judgments interacts with what they perceive as successful interventions. Most importantly, if the problem was thought to have a social cause (bullying) and/or when it was thought to be environmentally treatable, the attribution of disorder decreased. There was no synergistic or dampening effect of these two variables, which largely seemed to have functioned independently (at least at the group analysis level we use here).

Before we explore interpretations of the results and their societal and theoretical relevance, we will list what we see as the most critical limitations of this study because it is essential to keep these in mind when the implications are discussed.

A first limitation is that the vignettes described a rather specific problem and quite specific causes and treatments. Hence, it remains an open question to what extent we can generalize to other conditions, causes and treatments. Follow-up studies should inquire whether other conditions are more easily seen as disorders when they are caused by genetics rather than by environmental factors. Relatedly, we did not investigate whether the perceived severity of the condition mediated the effect of perceived causes and treatments on disorder attribution. Not surprisingly, the severity of conditions positively correlates with the extent to which they are seen as typical diseases (Hofstad et al., 2020). It would be interesting to know whether, for instance, the participants in our study assumed that pills are prescribed for more severe conditions. This effect seems at least a good explanation for why consulting a doctor strengthens the tendency to see the problem as a disorder.

A second limitation has to do with the study's ecological validity. There seems to be a consensus among medical researchers and physicians that both genes and environmental factors contribute to almost all observed interindividual variation. This is especially true for those conditions that are considered developmental. Likewise, general practitioners, paediatricians, and child psychiatrists will often prescribe both psychostimulants and behavioural therapy in cases of severe concentration difficulties (Pliszka & AACAP Work Group on Quality Issues, 2007). Many medical professionals would consider our presentation of the 'causes' and 'cures' rather ‘reductionist’ or oversimplified. Still, some research suggests that the multifactorial view is not widely accepted by the general public (Angermeyer & Dietrich, 2006), and many doctors only prescribe psychostimulants for concentration problems (Bachmann et al., 2017; Wolraich et al., 2019). Moreover, qualitative research from one of the authors has pointed out that, at least in Belgium, diagnosticians often explain another developmental disability, autism, by referring to neurological or genetic differences (Hens & Langenberg, 2018). Anecdotal evidence suggests that they may do so because they believe it helps acceptance of one's challenges. It is relatively safe to assume that similar practices occur with attention difficulties. Hence, although splitting up causes or cures may seem overly simplistic and reductionist, it is probably the way many laypeople and perhaps medical professionals think.

A third limitation is that most of the respondents were from the Global North. How non-Westerners conceptualize disorders may differ substantially from the Western disorder concepts, as medical anthropologists have claimed (Foster, 1976; Raguž & Alebić, 2021). Moreover, it seems likely that the link between genes and the realness of a phenomenon is typically Western. Fourthly, the observed effects of cause and treatment on disorder perception are statistically significant, but the effects are not very strong, hiding sizeable individual variation. Fifthly, our use of the word ‘disorder’ rather than ‘disease’ may imply that the results are not generalizable to disease judgments, and follow-up studies are required to check whether using the term ‘disease’ in this context would make a difference. Sixthly, the name Jada is gendered and may be associated with a specific ethnography, which may have influenced the results. Yet, a pilot indicated that vignettes in which we used Jason instead of Jada made little or no difference in the results. Most of these limitations pertain to the replicability of the findings. We call for further research exploring the replicability of the effects of causes, treatments, and their effectiveness with other problems, in cases where double causes or double treatments are described, with non-western populations, with other attributions (disease, disorder), and with other concrete variations like name, gender and ethnic connotation of the protagonist.

Bearing these limitations in mind and pending further corroborations, we propose that a plausible interpretation of our results is that the tendency to call attention problems disorders decreases (a) if this condition is thought to have a social cause (broadly construed) or (b) if the person is prescribed an environmental treatment. Also, our results suggest a main effect of treatment effectiveness on disorder judgments, whereby the success of pharmacological interventions decreases the likelihood that the person still has a disordered condition after the (successful) treatment more than a successful environmental treatment. Perhaps this is because attention problems are already quite ‘pathologized’, ‘geneticized’ and ‘pharmacologized’. Participants may have assumed that if a person has attention problems, these problems are genetically caused and should be treated pharmacologically. In this respect, it is noteworthy that disorder perception is high in the baseline conditions. Most laypeople and medically trained professionals believe medication is most effective for biologically caused diseases (Ahn et al., 2009-3). Quite possibly, the participants interpreted the prescription of the pharmacological intervention as further evidence for the biological base of the condition and hence for the 'realness' of the disorder (Lebowitz & Appelbaum, 2019). Relatedly, when the attention difficulties disappear after taking a pill, participants seem to think that the disorder is more likely to be cured than when successful treatment involves an intervention in the educational environment. This can be interpreted as indicating that environmental intervention is thought to reduce the symptoms. In contrast, pharmacological intervention is believed to impact the disorder (and its underlying mechanisms?). This is especially interesting, given that diagnoses of attention disorders are purely based on behavioural assessments and that it is thus unclear whether there can still be a disorder after the 'symptoms' have disappeared.

A demonstrable genetic ‘cause’ for mental, psychological and behavioural phenomena does normative work (Haslam et al., 2007): it seems to transform a problem into a disorder worthy of help and medical care. Hence, for those who want their difficulties and problems to be taken seriously, linking their complaints to a (likely) genetic cause or pharmacological treatment has certain benefits. On the other hand, as Kidd and Carel have suggested, a demonstrable cause as a requirement for disease attribution risks neglecting the importance of experienced suffering (Kidd & Carel, 2019; Kidd & Carel, 2017). To refer back to the vignettes we used, the problems that Jada experiences after being bullied may very well cause her as much suffering as genetically caused problems would, but she is less likely to receive medical treatment or even acknowledgement for her issues.

Furthermore, the link between a genetic cause and the effectiveness of pharmacological treatment is far less clear than what our respondents seem to intuit. After all, *ex juvantibus*-reasoning can sometimes be fallacious. Relatedly, although binary relations between genetic cause and pharmacological treatments seem intuitively straightforward, holistic approaches to health, as suggested by systems biology approaches, challenge these conceptions.

This last point ties in with some medicalization critiques in the sociology of medicine (Conrad, 2007). In that literature, claims are made about causal links between three relatively recent developments in the Global North, trends that are sometimes subsumed under the concept of ‘biomedicalization’ (Clarke et al., 2021). These trends, which we already hinted at earlier in this section, are (1) medicalization, the process of seeing more and more aspects of human life as pathological and as medical problems (Conrad, 2007), (2) geneticization, the tendency to define and understand interindividual differences in genetic terms (Phelan, 2005), and (3) pharmaceuticalization, “the process by which social, behavioural or bodily conditions are treated, or deemed to require treatment/intervention, with pharmaceuticals by doctors, patients, or both” (Abraham, 2010, p. 290; see also Maturo, 2012). Both philosophers and sociologists of medicine believe these trends are real and strengthen each other, although it is also acknowledged that the relations between them are complex (Abraham, 2002; Bell & Figert, 2012; Hedgecoe, 1998). The results of our study support the claim that these factors contribute to the perception of disorder, but we did not find that cause and treatment interact.

Medical sociologists also often consider medicalization decisively undesirable. The results of this study may prove helpful for developing campaigns and other policies to counteract the medicalization of ordinary thinking and healthy behaviour. After all, reforming an undesirable phenomenon usually requires a detailed description of factors that generate and stabilize the phenomenon. Sometimes the solution for incorrect medicalization was thought to come from philosophical analyses of illness and disease concepts (Boorse, 1997; Wakefield, 1992). These philosophical analyses were and are still used to argue against the medicalization of allegedly normal or healthy conditions (De Block & Adriaens, 2013; Horwitz & Wakefield, 2007). Nevertheless, there is considerable dissensus about what constitutes a correct analysis of disease and disorder and whether such analyses are even possible.

Moreover, it remains an open question to what extent philosophical analyses and arguments can convince non-philosophers (Schwitzgebel & Cushman, 2012). Be it as it may, the results of this study seem relevant for such analyses, and they can certainly be part of ‘naturalized conceptual analyses' (Machery, 2017) by making explicit which inferences concepts such as ‘disorder’ and ‘gene’ underwrite. In fact, work in this spirit has already been done on the concept of innateness (Griffiths et al., 2009) and, indeed, the gene concept (Stotz et al., 2004). Many of these inferences may be flawed, for instance, the inference that if a condition is a disorder, it probably has a ‘biological’ cause. Nevertheless, making these inferences explicit can help resist them if deemed necessary or desirable. Relatedly, how clinicians think about and use disorder concepts has played a role in decisions about nosography, and philosophers employing traditional conceptual analysis have occasionally set up empirical studies to see how well their analyses predicted judgments that involve the analyzed concept(First et al., 2018). For instance, Jerome Wakefield relied on his harmful dysfunction analysis of disorder (and disease) to predict that professionals' and lay judgments about (conduct) disorder are guided by his dysfunction requirement. This hypothesis was supported by the results of several studies (Kirk et al., 1999; Wakefield, 2006). Our study was not a direct attempt to test extant conceptual analysis of disease or disorder. However, it shows how the raw material of these analyses - our intuitive disorder judgments - seems to be influenced by factors usually neglected in philosophical accounts of disease and health. At the very least, we demonstrated that factors contributing to folk attributions of disorder may not solely be related to experienced suffering or objectively measurable deviations from an evolved (Matthewson & Griffiths, 2017) or statistical norm. Indeed, it seems that how cures and treatments are presented influences disorder attribution as well. Given the relation of disorder attribution with important societal and policy issues, this should give us pause.

## Author statement

ADB and KH designed the protocol and the vignettes. SDW performed the statistical analysis. All authors contributed equally in writing the paper.

## Figures and Tables

**Fig. A.1:**
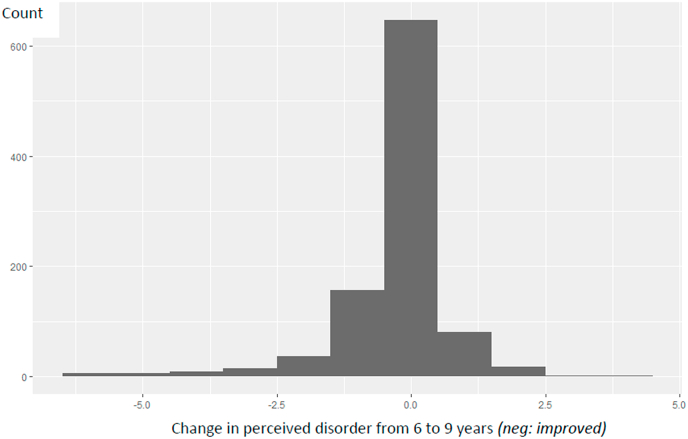
The distribution of change in disorder perception from age 6 to 9.

## Data Availability

Data will be made available on request.

## Appendix 1

As a preliminary step, we analyzed the overall change in disorder attribution from age six to nine. Fig. A.1 shows that the majority (66.6% of the 972 observations) when asked about disorder attribution at age 9, stuck to the perception they had for age six.

## Footnotes

1 The study was approved by the KU Leuven Social and Societal Ethics Committee (SMEC) on March 21, 2021, file number: 2021-3189-R2(MAR).

2 The preregistration of this study can be found here: https://osf.io/4mzg7

3 As these are often associated with challenges related to attention.

4 Appendix 1 shows the distribution of the change. Although many stuck to their original assessment, a sufficient number of participants shifted to allow an analysis.
